# Dysregulation of Cytosolic c-di-GMP in *Edwardsiella piscicida* Promotes Cellular Non-Canonical Ferroptosis

**DOI:** 10.3389/fcimb.2022.825824

**Published:** 2022-02-04

**Authors:** Ying Wen, Ying Wang, Shouwen Chen, Xiangshan Zhou, Yuanxing Zhang, Dahai Yang, Gabriel Núñez, Qin Liu

**Affiliations:** ^1^ State Key Laboratory of Bioreactor Engineering, East China University of Science and Technology, Shanghai, China; ^2^ Department of Pathology and Comprehensive Cancer Center, University of Michigan, Ann Arbor, MI, United States; ^3^ Southern Marine Science and Engineering Guangdong Laboratory (Zhuhai), Zhuhai, China; ^4^ Shanghai Engineering Research Center of Maricultured Animal Vaccines, Shanghai, China

**Keywords:** *Edwardsiella piscicida*, c-di-GMP accumulation, non-canonical ferroptosis, bacterial virulence, pathogen infection

## Abstract

Programmed cell death plays an important role in modulating host immune defense and pathogen infection. Ferroptosis is a type of inflammatory cell death induced by intracellular iron-dependent accumulation of toxic lipid peroxides. Although ferroptosis has been associated with cancer and other sterile diseases, very little is known about the role of ferroptosis in modulating host-pathogen interactions. We show that accumulation of the secondary messenger bis-(3′,5′)-cyclic dimeric GMP (c-di-GMP) in the pathogenic bacterium *Edwardsiella piscicida* (*E. piscicida*) triggers a non-canonical ferroptosis pathway in infected HeLa cells. Moreover, we observed that the dysregulation of c-di-GMP in *E. piscicida* promotes iron accumulation, mitochondrial dysfunction, and production of reactive oxygen species, all of which that can be blocked by iron chelator. Importantly, unlike classical ferroptosis that is executed *via* excess lipid peroxidation, no lipid peroxidation was detected in the infected cells. Furthermore, lipoxygenases inhibitors and lipophilic antioxidants are not able to suppress morphological changes and cell death induced by *E. piscicida* mutant producing excess c-di-GMP, and this c-di-GMP dysregulation attenuates bacterial virulence *in vivo*. Collectively, our results reveal a novel non-canonical ferroptosis pathway mediated by bacterial c-di-GMP and provide evidence for a role of ferroptosis in the regulation of pathogen infection.

## Introduction

Programmed cell death is comprised of non-lytic, immunologically silent forms of cellular demise such as apoptosis and lytic and pro-inflammatory forms of necrosis that include pyroptosis, necroptosis, and ferroptosis (Jorgensen et al., 2017). Ferroptosis is a more recently described modality of regulated necrosis, which is triggered by intracellular iron accumulation and excess peroxidation of polyunsaturated fatty acids through nonenzymatic reactions and lipoxygenase (LOX)-dependent enzymatic mechanisms (Dixon et al., 2012; Hassannia et al., 2019). Ferroptosis is regulated by glutathione peroxidase 4, a lipid repair enzyme capable of detoxifying hydroperoxides in complex lipids, and is induced by the inhibition or loss of glutathione peroxidase 4 activity (Dixon et al., 2012; Hassannia et al., 2019). A key feature of ferroptosis is that it can be blocked by iron chelators and lipophilic anti-oxidants (Dixon et al., 2012; Yang et al., 2014; Hassannia et al., 2019; Li et al., 2020). However, the specific molecular mechanisms that mediate ferroptosis remain poorly understood.

Increasing evidence has demonstrated the important dual function of programmed cell death in modulating host immune defense and pathogen infection. The death of an infected cell promotes the elimination of intracellular pathogen and the release of intracellular contents including cytokines and DAMPs, which further contributes to pathogen clearance and host immune defense. For instance, the intracellular host pattern recognition receptor NLRC4 can sense bacterial flagellins of various bacteria including *Legionella pneumophila* (Katagiri et al., 2012), *Listeria Monocytogenes* (Cervantes et al., 2008; Warren et al., 2008), *Burkholderia thiailandensis* (Miao et al., 2010), and *Salmonella* typhimurium (Yang et al., 2013) triggering caspase 1-mediated pyroptosis and secretion of the inflammatory cytokines IL-1β and IL-18 which enhances immune responses and limits bacterial invasion. Conversely, pathogens have evolved many strategies to manipulate cell death for successful infection. For example, *Yersinia pestis* prevents inflammasome activation by secreting YopK to interfere with pathogen recognition by NLRP3 and NLRC4, limiting host cell death and pathogen clearance (Dewoody et al., 2011). Thus, the question of whether and how cell death performs a physiologic function during infection is a key to understand pathogen-host interaction and bacterial pathogenesis, and develop therapeutic approaches to control infection. The role of apoptosis, pyroptosis and necroptosis in the regulation of host-pathogen interactions has been extensively studied (Labbe and Saleh, 2008; Ashida et al., 2011; Jorgensen et al., 2017; Imre, 2020) The role of ferroptosis in cancer and other sterile conditions such as neurodegeneration and acute kidney injury has been reported (Li et al., 2020). However, very little is known about the function of ferroptosis during pathogen infection.

The intracellular marine pathogen *Edwardsiella piscicida* (*E. piscicida*) infects a wide range of hosts from fish to birds, reptiles, and humans (Mohanty and Sahoo, 2007; Leung et al., 2012). *E. piscicida* causes a range of diseases, from ascites to immune organ dysfunction as well as persistent systemic infection in fish (Leung et al., 2019). Upon infection, host NLRC4 and NLRP3 can sense *E. piscicida* type III secretion system needle protein leading to the activation of caspase 1-mediated inflammasomes and triggering pyroptosis, which promotes host immune defense (Chen et al., 2017). Moreover, *E. piscicida* Trxlp promotes NLRC4 inflammasome activation in macrophages (Xu et al., 2018) while hemolysin provokes non-canonical inflammasome activation and pyroptosis by promoting intracellular sensing of LPS in mammals and fish (Chen et al., 2018; Wen et al., 2019). Conversely, *E. piscicida* has evolved diverse strategies to inhibit cell death and evade host immune recognition and killing. For instance, *E. piscicida* inhibits NLRP3 inflammasome activation *via* a type VI secretion system effector EvpP by regulating intracellular Ca^2+^ influx and JNK phosphorylation (Chen et al., 2017). Intracellular *E. piscicida* was also found to alter its arginine metabolism to facilitate cytosolic spermine accumulation and ultimately block NLRP3 inflammasome activation and pyroptosis (Jiang et al., 2021). Moreover, the type III secretion system effector EseJ of *E. piscicida* was reported to inhibit apoptosis by down regulating type 1 fimbriae during infection (He et al., 2020). Collectively, these observations suggest that regulation of cell death is important during *E. piscicida* infection. However, the existence of additional cell death events during pathogen infection remains largely unknown.

Here we report that dysregulation of diguanylate cyclase (DGC) in *E. piscicida* induces host cell death with rupture of the cell membrane which is associated with attenuated virulence in zebrafish. We show that the activation of DGC results in elevated bis-(3′,5′)-cyclic dimeric GMP (c-di-GMP) in *E. piscicida* triggering an iron-dependent, oxidative ferroptosis, which is independent of lipid oxidation. Our results indicate that tight regulation of intracellular c-di-GMP is critical for infection and suggest the existence of a non-canonical ferroptotic pathway independent of lipid peroxidation that can be triggered by pathogen infection.

## Materials and Methods

### Bacterial Strains and Growth Conditions

The wild-type *E. piscicida* EIB202 (CCTCCM208068) has been described previously (Wang et al., 2009). *E. piscicida* 1906I was screened from the *E. piscicida* EIB202 transposon insertion mutant library (Yang et al., 2017) *E. piscicida Δ1906* strain was constructed by unmarked depletion of gene *ETAE_1906* in EIB202 strain while *E. piscicida* 1906I *Δ1905* was constructed by depletion of gene *ETAE_1905* in mutant 1906I. All the *E. piscicida* strains were inoculated at 1:100 and cultured statically for 12 h in tryptic soy broth (TSB) containing 16.7 μg/ml colistin at 30°C. *Escherichia coli* DH5α, cc118 λpir, SM10 λpir, and Stbl3 strains were used for the construction of plasmids and the conjugation of the *pir*-dependent suicide plasmid. *E. coli* strains were grown in Lysogeny broth or agar at 37°C.

### Preparation of *E. piscicida* Mutants

A previous in-frame gene mutation and deletion method based on allelic exchange was used to generate specific gene-deletion mutants of *E. piscicida* (Edwards et al., 1998). The upstream and downstream fragments of each indicated gene were fused by overlapping PCR and cloned into the *sacB* suicide vector pDMK and subsequently linearized with *Bgl*II and *Sph*I. Then the resulting plasmids were transformed into *E. coli* CC118 λ*pir*. The correct plasmids were then transformed into *E. coli* SM10 λ*pir* and conjugated into EIB202. The single-crossover mutants in which the plasmids have integrated into the chromosome by homologous recombination, were selected on tryptic soy agar plates containing kanamycin (50 μg/ml) or colistin (16.7 μg/ml). Then tryptic soy agar plates containing 10% sucrose were used to screen for the deletion mutants that completed allelic exchange. PCR amplification of the respective DNA loci and DNA sequencing of each PCR product were used to confirming the mutants. Primer pairs for generating upstream and downstream fragments of gene *ETAE_1906* or *ETAE_1905* are listed in **Table S1**.

### Generation of Mlkl-Knockout Cell Lines

Lentiviral CRISPR/Cas9 targeting guide RNA expressing vector (lentiCRISPRv2) was obtained from Addgene (#52961). The *mlkl* knockout target sequence used was 5’-GCATATTATCACCCTTGGCC-3’ (design by: http://zifit.partners.org). To generate gene knockout cell line, firstly lentiCRISPRv2 harboring the gene target sequence was constructed and transfected into 293T cells together with packing plasmids Δ8.91 and VSVG and with a ratio of 5:3:2. The supernatant of 293T cells which containing lentiviral particles was collected 48h after transfection and then was used to infect HeLa cells for another 48 h. Puromycin-resistant cells were then diluted to 5 cells/mL and seeded to 96-well plates, followed by a several-days expansion to establish a new clonal cell line. The clones with *mlkl*-deficiency were identified by immunoblotting with anti-MLKL antibody.

### Cell Culture and Infections

Wild-type HeLa, 293T, and NCI-H226 cells were all obtained from ATCC. The *mlkl*-deficient HeLa cell lines were generated as above, and *gsdmd*-deficient HeLa cells were kindly provided by Dr. Feng Shao (National Institute of Biological Sciences, Beijing). HeLa and 293T cells were cultured in Dulbecco’s Modified Eagle’s medium (Gibco) while NCI-H226 cells were cultured in RPMI 1640 medium (Gibco). All medium were supplemented with 10% (vol/vol) fetal bovine serum (Gibco) and 1% penicillin-streptomycin (Sigma-Aldrich). All cells were grown at 37°C in a 5% CO_2_ incubator.

For bacterial infection, cells were seeded and grown overnight to a density of 1×10^5^ cells per well of 24-well plates. Then the culture medium was replaced with phenol red-free Opti-MEM (Gibco) medium and cells were incubated with indicated *E. piscicida* strains at a multiplicity of infection (MOI) of 25. Infection was then initiated by centrifuging the plate at 600× *g* for 10 min and incubating at 35°C in an atmosphere containing 5% CO_2_. The time point after centrifugation was recorded as the 0 h post infection. To induce secondary pyroptosis or ferroptosis, cells were treated with 20 µM staurosporine (Sigma-Aldrich) or 20 µM RSL3 (MCE), respectively.

For caspase inhibitor assays, z-VAD-fmk (50 µM; MedChemExpress (MCE)), z-YVAD-fmk (50 µM; Sigma-Aldrich), z-VDVAD-fmk (50 µM; APExBIO), z-DEVD-fmk (50 µM; APExBIO), z-WEHD-fmk (20 µM; APExBIO), z-VEID-fmk (50 µM; APExBIO), z-IETD-fmk (50 µM; APExBIO), or z-LEHD-fmk (50 µM; APExBIO) were incubated, respectively, with cells at 37°C for 1 h before bacterial addition. For other pharmacological inhibitors assays, cells were pretreated with cytochalasin D (2 μg/ml; Invitrogen), azathioprine (50 µg/ml; Sigma-Aldrich), sulfathiazole (100 µg/ml; Sigma-Aldrich), Necrostatin-1 (Nec-1, 50 µM; MCE), GSK’872 (3 µM; MCE), necrosulfonamide (NSA, 20 µM; MCE), deferoxamine mesylate (DFM, 50 µM; MCE), PD146176 (5 µM; Sigma-Aldrich), zileuton (5 µM; MCE), ML351 (10 µM; Cayman), ferrostatin-1 (Fer-1, 20 µM; MCE), or liprostatin-1 (Lipro-1, 20 µM; MCE), respectively, for 30 min before being exposed to bacteria or other chemicals.

### Cell Death Assay

As described previously, a qualitative method based on cellular morphological observation and a quantitative method detecting the release cytosolic contents were used to examine cell death, respectively (Wen et al., 2019). Cells were treated as described previously in 24-well plates and the dynamic morphological changes of cells were captured using an Olympus IX71 fluorescent microscope at room temperature. Propidium iodide (PI, 5 ng/ml) was added to the medium as an indicator of cell membrane integrity. Quantitative cytotoxicity was detected by measuring lactate dehydrogenase (LDH) release of infected cells. The culture medium was collected at the indicated time points after bacterial infection and centrifuged at 600× *g* for 5 min. Then 50 ul clear supernatants were transferred to per well of 96-well plate (flat bottom) for subsequent LDH detection with a CytoTox 96 assay kit (Promega) according to the manual. Each sample was examined in triplicate and the absorbance at 490 nm was measured using a microplate reader (Dynex Technologies). The absorbance of supernatant from uninfected/untreated cells was used for background subtraction. Cytotoxicity was normalized to lysis buffer treatment (100% of control). The percentage of cell death was calculated as follows: Percentage (%) of cell death = (A490 nm of samples − A490 nm of uninfected cells)/(A490 nm of lysis buffer-treated cells − A490 nm of uninfected cells) × 100.

### Zebrafish Infection

The protocol for all the experiments on zebrafish here was approved by the Institutional Animal Care and Use Committee of East China University of Science and Technology (No. 2006272). Single colony of indicated *E. piscicida* strains was inoculated into TSB medium containing 16.7 μg/ml colistin and cultured overnight at 30°C in a shaking incubator with speed of 200 rpm, then inoculated at 1:100 into fresh pure TSB culturing statically at 30°C to an OD_600_ of 0.8. Bacteria was pelleted by centrifugation (4500× *g*) at 4°C for 10 min and washed three times with cold PBS, then suspended in PBS to 5000 CFU/ml. Zebrafish (6 months old) were randomly selected and intramuscularly injected with 10 μl bacterial suspension (50 CFU per fish) or PBS alone. Subsequently, the fish were transferred to clear tanks with a density of no more than 35 fish per tank and incubated at room temperature. The mortality was then recorded at the different time points. For bacterial colonization determination, five surviving zebrafish were pooled as a sample and dissected at the indicated post infection time points. Liver, spleen, kidneys, and intestines were collected and homogenized for CFU counts.

### Quantitative RT-qPCR

For RT-qPCR, total bacterial RNA was isolated by using an RNA isolation kit (Tiangen) according to the instruction manual and concentration of extracted RNA was measured by a NanoDrop spectrophotometer (Thermo Scientific). Then FastKing One Step RT-PCR Kit (Tiangen) was used for RNA reverse transcription and cDNA synthesis. The kit SuperReal Pre-Mix Plus (SYBR Green; Tiangen) was used for quantitative real-time PCR, which was performed on a FTC-200 detector (Funglyn Biotech). All the procedures followed the instruction manuals. Gene expression was evaluated for three biological replicates, and the data for each sample were calculated relative to the expression level of the 16S gene by using the 2^-ΔΔCT^ method. Primers used in this study are listed in **Table S2**.

### Bacterial c-di-GMP Detection

To quantitatively measure c-di-GMP level in *E. piscicida* strains, Cayman’s cyclic di-GMP ELISA kit was used. All the indicated strains were cultured as described above. Bacteria was cultured statically for 12-16 h and then pelleted by centrifugation (8000× *g*, 5 min) at 4°C, followed by triple wash with cold PBS to eliminate any organic components from culture medium. 8×10^9^ bacterium were collected at final as for each strain and 500 μl B-PER reagent (Thermo Fisher) was added to and mixed sufficiently with bacteria by pipetting. Incubating 15 mins at room temperature to lyse the bacteria, then clear supernatant of lysate was collected by centrifugation (15000× *g*) at 4°C for 10 min and kept on ice for following steps. Protein in the lysate supernatants was determinized by Pierce BCA protein assay (Thermo Scientific) and c-di-GMP was quantitatively measured with cyclic-di-GMP ELISA kit according to the manufacturer’s manuals. Bacterial c-di-GMP level was normalized to total protein amount of lysate supernatant.

### Detection of Bacterial Biofilm Formation and Exopolysaccharides (EPS) Production

To measure bacterial biofilm formation, the indicated *E. piscicida* strains were cultured overnight in TSB containing colistin at 30°C in a shaking incubator with speed of 200 rpm. Then 10 µl bacterial suspension in PBS with a concentration of 1×10^9^ CFU/ml was added into 200 µl TSB in each well of sterile 96-well polystyrene plates. There were 6 replicates for each strain. Bacteria was cultured at 30°С for 24 h. After that the culture fluid with planktonic bacteria was aspirated, and the wells were washed 3 times with PBS. The formed biofilm was fixed with 200 µl methanol for 15 min and stained by adding 200 µl of 0.1% crystal violet water solution into each well for 30 min at room temperature. Unbounded dye was removed by triple washing with PBS. To extract the dye bound with the adsorbed biofilm, 200 µl 95% ethanol was added to wells for 30 min and then absorbance at 590 nm of mixture was measured by a plate reader. A mixture of sterile PBS (10 µl) and TSB (200 µl) was used as the negative control for which the sequence of operations was same as for experimental wells, and the absorbance at 590 nm was used as a background subtraction. The relative biofilm formation ability was the ratio of the absorbance at 590 nm of indicated strains to that of wild-type strain EIB202.

The bacterial EPS production was quantified by Congo red binding assay. Overnight-cultured *E. piscicida* was collected and suspended with PBS with a concentration of 1×10^9^ CFU/ml and 2 ml bacterial broth were pelleted by centrifugation (4500× *g*, 10 min) at 4°C, followed by suspending and incubating with 1 ml M9 medium containing 40 µg/ml Congo red at 30°C in a shaking incubator with speed of 180 rpm for 3 h. Then, supernatant was separated with bacteria by centrifuging at 8000× *g* for 5 min. Aliquots of clear supernatant were transferred into a 96-well plate and the absorbance at 490 nm was measured. The absorbance of M9 medium alone was used as the background and should be subtracted. The absorbance of M9 medium containing 40 µg/ml Congo red was used as 100% of control. The bacterial EPS production positively correlated with Congo red adsorbed by bacteria which was calculated as following formula: Percentage of Congo red adsorption by bacteria = (1- *A*_samples_/*A*_control_) × 100%.

### Assessment of Bacterial Adhesion and Internalization

HeLa cells were seeded into 24-well plate with density of 1×10^5^ cells per well and infected with indicated *E. piscicida* strains at a MOI of 100 for 1 h. Followed by that, cells were washed with cold PBS for 3 times to remove the unattached bacteria. To assess bacteria adhesion, cells were then lysed with 1% Triton X-100 and the cell lysate containing attached bacteria was serially diluted and CFU was counted. The ability of bacterial adhesion was calculated as the ratio of CFU per cell. To assess bacterial internalization, cells were incubated with Opti-MEM containing with 800 μg/ml streptomycin for 0.5 h to kill extracellular bacteria and then washed with cold PBS for another 3 times followed by lysis with 1% Triton X-100. The cell lysate containing internalized bacteria was serially diluted and CFU was counted. The ability of bacterial internalization was calculated as the ratio of CFU per cell.

### Caspase Activity Assays

The activity of caspases was assayed according to the substrate manufacturer’s instructions. Briefly, HeLa cells were infected with *E. piscicida* EIB202 or 1906I for 3.5 h and collected by trypsin-digesting and centrifuging (600× *g*, 5 min, 4°C), then lysed with lysis buffer provided in the caspase activity assay kit. Subsequently, chromogenic substrates (Beyotime Biotechnology) of human caspase-1 (Ac-YVAD-pNA), caspase-2 (Ac-VDQQD-pNA), caspase-3/7 (Ac-DEVD-pNA), caspase-4 (Ac-LEVD-pNA), caspase-6 (Ac-VEID-pNA), caspase-8 (Ac-IETD-pNA), and caspase-9 (Ac-LEHD-pNA) were incubated with cell lysates at a final concentration of 200 µM, while a fluorescent substrate Ac-WEHD-AFC (BioVision) was used to detect the activity of caspase-5 in lysate, which has an excitation at 400 nm and emission at 505 nm. Caspase activity was examined by measuring changes in absorbance at 405 nm or fluorescence at 505 nm at 5-min intervals caused by the presence of free pNA hydrolyzed from the substrates. Uninfected HeLa cells was taken as a negative control and background subtraction. The relative caspase activity was the absorbance or fluorescence of cells infected with 1906I relative to the absorbance or fluorescence of EIB202 samples.

### Antibodies

All antibodies were used for Western blotting. Rabbit anti-MLKL monoclonal antibody (1:1000; Abcam) was used to verify clones with MLKL deficiency while rabbit anti-GSDME-N-terminal monoclonal antibody (1:1000; Abcam) was used for detecting cleavage of GSDME. Mouse anti-β-actin monoclonal antibody (1:1000; Cell Signaling Technology) was used to indicate the loaded protein amount.

### Determination of Intracellular Labile Iron

Fluorescent probes Calcien-AM (CA-AM, Invitrogen) and Phen green SK (PGSK, Invitrogen), which bind iron rapidly, stoichiometrically, reversibly while performing fluorescence-quenched complexes (Epsztejn et al., 1997; Petrat et al., 1999), were used to quantitively measure cytosolic labile iron. Briefly, 1×10^5^ HeLa cells were seeded into 24-well plate for overnight culture and incubated with DFM-added Opti-MEM or Opti-MEM alone at 37°C for 30 min before infection of *E. piscicida* EIB202 or 1906I, or treatment of 200 µM ferric ammonium citrate (FeAC). Control cells were only incubated with Opti-MEM. At 3 h post incubation of bacteria or FeAC, cells were rinsed once with PBS and then incubated with Opti-MEM containing 250 nM CA-AM or 20 µM PGSK at 37°C for 20 min, respectively. The CA-AM and PGSK are non-fluorescent lipophilic, esterified precursors and rapidly permeate the plasma membrane. Once inside the cell, they are hydrolyzed to be fluorescent iron chelator. The fluorescence is then quenched immediately by intracellular iron. Excess CA-AM or PGSK that did not penetrate the cells was removed by triple wash with PBS and 500 µl Opti-MEM was added to each well. Subsequently, CA-AM or PGSK fluorescence intensity of cells were measured with a Multiscan Spectrum microplate reader (Synergy H1, BioTek) at an excitation wavelength of 485 nm and an emission wavelength of 530 nm. The quenched fluorescence intensity compared to untreated control cells reflected the cytosolic labile iron accumulated in cells after bacterial infection or FeAC treatment.

To measure labile iron in mitochondrial, cells were incubated with mitochondria-targeted fluorescence probe Mito-FerroGreen (Dojindo) in accordance with the manufacturer’s instructions. Briefly, after appropriate treatments with DFM or infection with bacteria, HeLa cells were washed three times with HBSS at 3.5 h post infection and incubated with 5 μM Mito-FerroGreen for 30 min at 37°C in a 5% CO_2_ incubator. Followed by discarding the supernatant, HeLa cells were washed three times with HBSS and then dissociated by adding enzyme-free cell dissociation buffer (Gibco) and collected for flow cytometry analysis. Sample data was collected utilizing a BD FACSCelesta flow cytometer. 10000 events were collected for each sample after gating out debris and fluorescence of cells were measured using F-1 FITC channel. Data files were analyzed using FlowJo V10. Fluorescence intensity represented the labile iron in mitochondrial.

### Mitochondrial Potential Assay (JC-1)

A fluorescent lipophilic carbocyanine dye JC-1 (MCE) was used to indicate changes of mitochondrial membrane potential (ΔΨm). JC-1 forms complexes known as J-aggregates at high ΔΨm which emits an orange-red fluorescence (Ex/Em=585nm/590nm). While in cells with low ΔΨm, JC-1 remains in the monomeric form emitting a green fluorescence (Ex/Em=510nm/527nm). Briefly, 1×10^5^ HeLa cells were seeded into 24-well plate and cultured overnight. Followed by appropriate treatments with DFM or infection with indicated *E. piscicida* strains, HeLa cells were washed three times with PBS at 3.5 h post infection and then incubated with Opti-MEM containing 2 µM JC-1 at 37°C for 20 min in a 5% CO_2_ incubator. After JC-1 staining, cells were washed three times with PBS and 500 µl Opti-MEM was added to the wells. Fluorescence of JC-1 aggregates and monomers were observed and images were directly photographed by Olympus IX71 fluorescent microscope under FITC and TRITC filter.

### Cellular ATP Detection

ATP level in cells with or without infection was quantitatively detected by using a luminescent ATP-based cell viability assay kit (Promega) according to the slightly modified instruction manual. Briefly, 1×10^5^ HeLa cells were seeded into 24-well plate and cultured overnight. Cells were pretreated with DFM or incubated with Opti-MEM alone at 37°C for 30 min and then infected with *E. piscicida* EIB202 or 1906I at a MOI of 25 for 3.5 h. Partial cell culture medium was removed leaving 200 µl medium in the well. Then an equivalent volume of CellTirter-Glo 2.0 Reagent from Promega kit was added into each well and incubated for 10 min on an orbital shaker to induce cell lysis and reaction at room temperature. Subsequently, 100 µl reaction buffer was transferred into an opaque-walled 96-well plate and luminescence was recorded which is proportional to the amount of ATP present in cells. The luminescence of mixture of CellTirter-Glo 2.0 Reagent with pure Opti-MEM was a background and should be subtracted when calculating luminescence of other samples.

### Mitochondrial and Cytosolic Reactive Oxygen Species (ROS) Measurement

A fluorescent mitochondrial-targeted probe MitoSOX (Invitrogen), oxidation of which by superoxide produces red fluorescence, was used to detect mitochondrial ROS production according to the instruction manual. Briefly, Cells were pretreated with DFM or incubated with pure Opti-MEM at 37°C for 30 min and then infected with *E. piscicida* EIB202 or 1906I at a MOI of 25 for 3.5 h. After that, HeLa cells were washed once with HBSS and incubated with 5 μM MitoSOX for 10 min at 37°C in a 5% CO_2_ incubator. Subsequently, cells were washed gently with warm HBSS for three times and then dissociated by adding enzyme-free cell dissociation buffer (Gibco) and collected for flow cytometry analysis. Sample data were collected utilizing a BD FACSCelesta flow cytometer. 10000 events were collected for each sample after gating out debris and fluorescence of cells were measured using F-2 PE channel. Data files were analyzed using FlowJo V10. Fluorescence intensity represented mitochondrial oxidative stress.

DCFH-DA fluorescence probe (Beyotime) was used to assess cytosolic ROS. Once DCFH-DA permeates the plasma membrane, it is then deacetylated by cellular esterases to a non-fluorescent compound, which is later oxidized by ROS into highly green-fluorescent DCF. Based on this principle, cells with stronger oxidative stress exhibit higher value of green fluorescence. For analysis, cells were washed once with PBS after 3.5 hours’ infection with *E. piscicida* strains and incubated with Opti-MEM containing 10 µM DCFH-DA at 37°C for 20 min in a 5% CO_2_ incubator. Subsequently, excess DCFH-DA outside the cells was removed by washing cells three times with Opti-MEM. Cell fluorescence was observed and images were captured with Olympus IX71 fluorescent microscope with laser at excitation wavelength 488 nm (FITC filter).

### Lipid Peroxidation Detection

A lipid-soluble fluorescent indicator C11-BODIPY^581/591^ (Invitrogen) was used to detect lipid peroxidation. Non-oxidized C11-BODIPY^581/591^ can be excited at wavelength of 581 nm and emits red fluorescence (Ex/Em=581nm/591nm). Upon oxidation, its excitation maximum shifts from 581 to 500 nm and the emission maximum shifts from 591 nm to 510 nm, emitting green fluorescence. Briefly, 1×10^5^ HeLa cells were seeded into 24-well plate and cultured overnight. Followed by appropriate treatments with RSL3 or infection with indicated *E. piscicida* strains, HeLa cells were washed three times with PBS at 3.5 h post infection or 20 µM RSL3 treatment and then incubated with Opti-MEM containing 2 µM C11-BODIPY^581/591^ at 37°C for 30 min in a 5% CO_2_ incubator. After staining, cells were washed three times with PBS and 500 µl Opti-MEM was added to the wells. Fluorescence of non-oxidized and oxidized C11-BODIPY^581/591^ were observed and images were photographed by Olympus IX71 fluorescent microscope with lasers under FITC and TRITC filter.

### Statistical Analysis

Data were presented as the mean ± standard deviation (SD) of triplicate sample per experimental condition unless noted otherwise. Representative results were shown in the figures. Statistical analyses were performed using two-way ANOVA, one-way ANOVA, multiple *t* test, and Gehan-Breslow-Wilcoxon test of GraphPad Prism Program (Graphpad Software). Error bars represent the standard deviation. Significant statistical differences were indicated by asterisks: *P < 0.05; **P < 0.01; ***P < 0.001.

## Results

### A Transposon Insertion in *E. piscicida* Promotes Lytic Cell Death in HeLa Cells and Reduces Pathogen Virulence in Zebrafish

**Figure 1:**
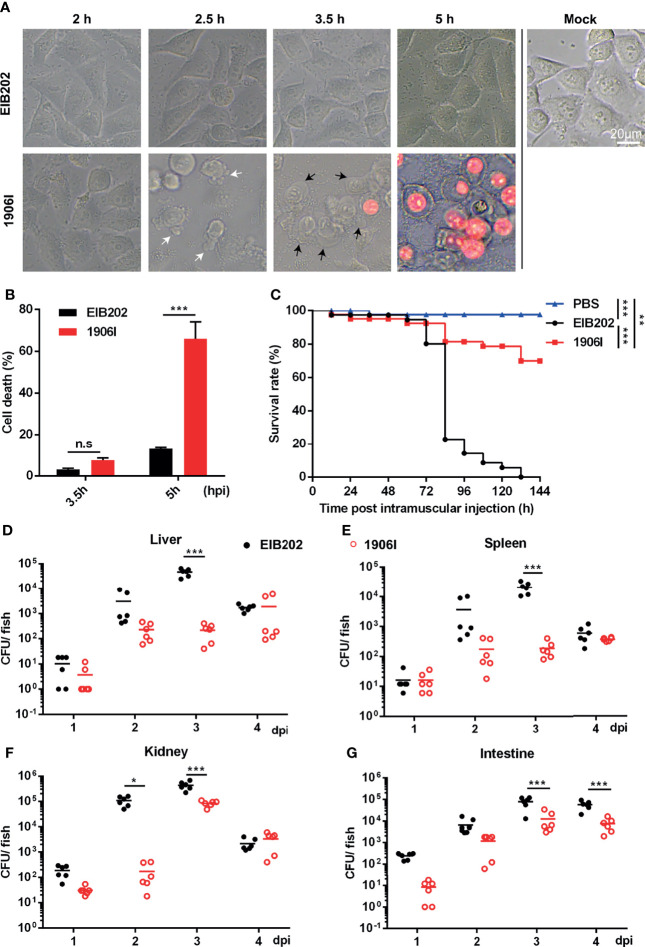
*E. piscicida* 1906I induced robust membrane-ruptured cell death and showed attenuated bacterial colonization and virulence in zebrafish. **(A)** Dynamic changes of HeLa cell morphology after infection of wild-type EIB202 or mutant 1906I at an MOI of 25 for the indicated time. White arrows denote the membrane bebbling and black arrows denote membrane swelling and rounding. Propidium iodide (PI) was added to detect the loss of plasma membrane integrity. Scale bar, 20 μm. **(B)** Lactate dehydrogenase (LDH) release of HeLa cells infected with EIB202 or 1906I for the indicated time. **(C–G)** Adult zebrafish were intramuscular injected with 50 CFU indicated *E. piscicida* strains per fish. **(C)** Survival of zebrafish was monitored for 6 days. PBS as control. (*n*=35). **(D–G)** Bacteria colonization in liver **(D)**, spleen **(E)**, kidney **(F)** and intestine **(G)** of the indicated time points post infection. Five surviving adult zebrafish were pooled as a sample and dissected at the indicated time point post infection, and livers, spleens, kidneys, and intestines were collected and grinded to homogenates for CFU counts, respectively. Results are representative of at least three independent experiments. *P < 0.05, **P < 0.01, ***P < 0.001; n.s, not significant. [Gehan-Breslow-Wilcoxon test for panel **(C)**, Two-way ANOVA for panels **(B, D–G)**].

To explore the physiologic function of cell death and the strategies used by pathogens to regulate host cell death during infection, we previously utilized a transposon insertion mutant library of *E. piscicida* to identify mutants that induce membrane-ruptured cell death based on cell morphology changes and LDH release (Chen et al., 2018; Wen et al., 2019). A novel *E. piscicida* mutant which was named 1906I was found to trigger cell death in HeLa cells upon infection. At the early period of infection (at 2.5 h post infection), HeLa cells infected with mutant 1906I began to exhibit some morphological features of apoptosis, such as membrane blebbing and formation of balloon-like protrusions (**Figure 1A**). Subsequently, 1906I-infected cells showed swelling and rounding, and began to show increased membrane permeability and loss of plasma membrane integrity by 3.5 h post infection. Cells exhibited membrane ruptured as the cytoplasm appeared red after incubation with the membrane-impermeable PI (**Figure 1A**). By contrast, the morphology of HeLa cells infected with wild-type *E. piscicida* EIB202 was similar to that of untreated cells (**Figure 1A**). Consistent with morphological changes shown with PI-positive staining, increased LDH release was detected in 1906I-infected HeLa cells during the late period of infection (at 5 h post infection) compared to that observed in EIB202-infected cells (**Figure 1B**). These results indicate that the transposon gene insertion in the *E. piscicida* 1906I mutant promotes robust cell death associated with ruptured membrane.

We next determined the function of the 1906I mutant after infection *in vivo*. To assess this question, we infected adult zebrafish with wild-type EIB202 or 1906I by intramuscularly injecting the fish with 50 CFU. We found that all zebrafish succumbed after infection with EIB202 whereas 1906I infected fish exhibited remarkably reduced mortality (**Figure 1C**). Consistent with reduced mortality, fish infected with 1906I mutant showed lower bacterial loads in the liver, spleen, kidney, and intestine on days 2 and 3 after infection than animals infected with wild-type EIB202 (**Figures 1D–G**). These results suggest that gene disruption by transposon insertion in the 1906I mutant limits *E. piscicida* colonization in zebrafish.

### Upregulation of Cytosolic c-di-GMP in *E. piscicida* Induces Robust Membrane Rupture-Mediated Cell Death

**Figure 2:**
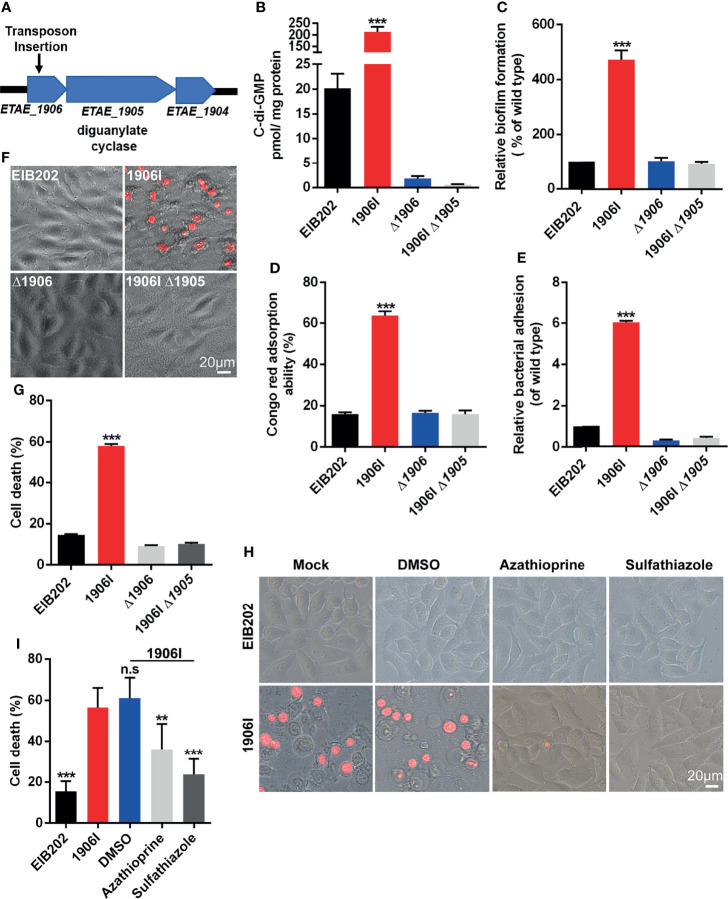
Upregulated c-di-GMP in 1906I strain promoted robust membrane-ruptured cell death. **(A)** A schematic of transposon insertion location in the genome of 1906I strain. **(B)** Cytosolic c-di-GMP levels of indicated *E. piscicida* strains. **(C)** Biofilm formation of indicated *E. piscicida* strains detected by crystal violet staining method. **(D)** Exopolysaccharide production indicated by Congo red adsorption of indicated *E. piscicida* strains. **(E)** Bacterial count by agar plating cell lysates from HeLa cells infected by indicated *E. piscicida* strains (MOI = 100, 1 hpi) after 3 times washing with cold PBS to remove unattached bacteria. **(F, G)** Cell morphology with PI staining **(F)** and LDH release **(G)** of Hela cells after infection with indicated *E. piscicida* strains (MOI=25, 5 hpi). Scale bar, 20 μm. **(H, I)** Cell morphology with PI staining **(H)** and LDH release **(I)** of HeLa cells infected with *E. piscicida* EIB202 or 1906I (MOI=25, 5 hpi) in the presence of c-di-GMP synthesis inhibitor azathioprine (50 µg/ml), sulfathiazole (100 µg/ml), DMSO. Scale bar, 20 μm. Results are representative of at least three independent experiments, and error bars denote SD of triplicate wells. **P < 0.01, ***P < 0.001; n.s, not significant. (One-way ANOVA).

*E. piscicida* is an intracellular pathogen, but the 1906I-induced cell death was found to be independent of intracellular bacteria since 1906I showed lower internalization ability and inhibitor of endocytosis displayed no suppressive effect on cell death (**Figure S1**). To further investigate the mechanism by which 1906I induces lytic cell death, we analyzed the transposon insertion site within 1906I and found that the transposon was inserted in an operon containing the gene *ETAE_1905* that encodes DGC (**Figure 2A**). DGC synthesizes the second messenger c-di-GMP from two GTP molecules which facilitates the biosynthesis of bacterial adhesins and EPS as well as biofilm formation by increasing c-di-GMP levels (Hengge, 2009). Bioinformatic analysis revealed the gene *ETAE_1906* inactivated by transposon insertion was located upstream of *Dgc*, the gene encoding DGC (*ETAE_1905*) (**Figure 2A**). Transcriptional analysis of genes adjacent to insertion site indicated significant upregulation of *ETAE_1905* in 1906I compared with the wild-type EIB202 (**Figure S2**). In agreement with upregulated transcription levels of *ETAE_1905*, 1906I produced higher c-di-GMP levels than EIB202 (**Figure 2B**). Consistent with increased levels of c-di-GMP, the 1906I mutant showed enhanced biofilm formation (**Figure 2C**), EPS production (**Figure 2D**) and adhesion to HeLa cells (**Figure 2E**). In contrast, deletion of *ETAE_1906* in EIB202 decreased slightly the c-di-GMP levels, but did not affect biofilm formation, exopolysaccharide production or adhesion to HeLa cells compared with EIB202 (**Figures 2B–E**). Importantly, deletion of *ETAE_1905* in the 1906I mutant greatly impaired c-di-GMP levels and the increased biofilm formation as well as the production of EPS, and adhesion to HeLa cells (**Figures 2B–E**). Furthermore, inactivation of *ETAE_1905* eliminated the ability of 1906I to induce cytotoxicity in HeLa cells (**Figures 2F, G**), suggesting the overexpression of *ETAE_1905* instead of disruption of *ETAE_1906* induced both bacterial phonotypes and cell death.

To further verify the role of DGC and c-di-GMP in the regulation of cell death, we treated HeLa cells with azathioprine or sulfathiazole, two small molecules that inhibit biosynthesis of c-di-GMP (Antoniani et al., 2010; Antoniani et al., 2013). Cell death and morphological changes induced by the 1906I mutant were inhibited by treatment with either azathioprine or sulfathiazole (**Figures 2H, I**). Collectively, these results indicate that the 1906I mutant overexpresses the gene encoding DGC leading to increased levels of c-di-GMP which is important for induction of lytic cell death in host cells.

### Dysregulated c-di-GMP-Induced Cell Death Is Not Mediated by Caspases or the RIP1/3-MLKL Axis

**Figure 3:**
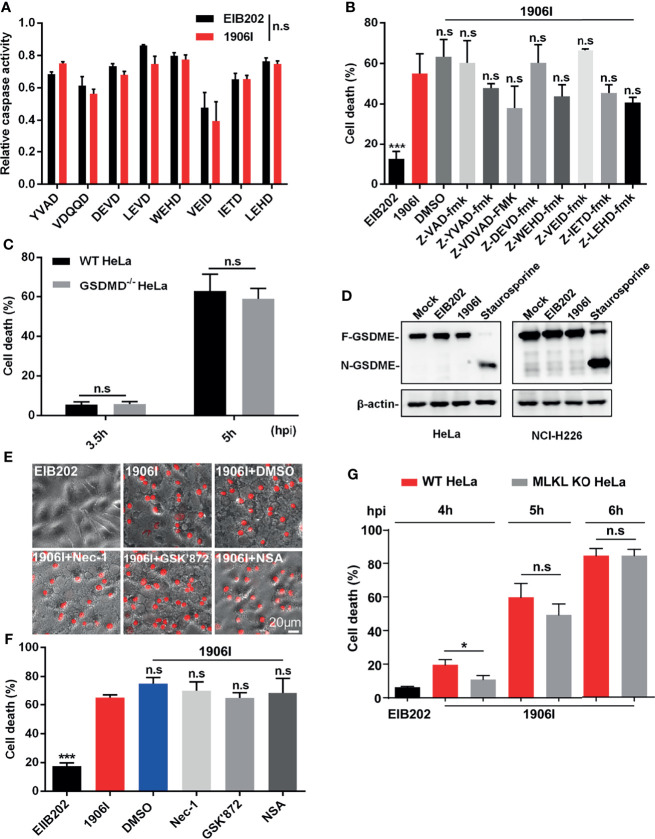
Cell death induced by c-di-GMP-upregulated *E. piscicida* was independent of caspases, Gasdermin D/E or RIP1/3-MLKL axis. **(A)** HeLa cells were infected with *E. piscicida* EIB202 or 1906I (MOI=25, 3.5 hpi). The relative caspase activity was then measured by incubating cell lysate of infected or uninfected cells with chromogenic substrates of caspase-1 (Ac-YVAD-pNA), caspase-2 (Ac-VDQQD-pNA), caspase-3/7 (Ac-DEVD-pNA), caspase-4 (Ac-LEVD-pNA), caspase-6 (Ac-VEID-AFC), caspase-8 (Ac-IETD-pNA) and caspase-9 (Ac-LEHD-pNA), or fluorogenic substrates of caspase-5 (Ac-WEHD-AFC). **(B)** HeLa cells were infected with *E. piscicida* EIB202 or 1906I at an MOI of 25 in the presence of z-VAD-fmk (50 µM), z-YVAD-fmk (50 µM), z-VDVAD-fmk (50 µM), z-DEVD-fmk (50 µM), z-WEHD-fmk (20 µM), z-VEID-fmk (50 µM), z-IETD-fmk (50 µM), z-LEHD-fmk (50 µM), or DMSO. LDH release was detected at 5 h post infection. **(C)** LDH release of wild-type or *gsdmd* ^-/-^ HeLa cells infected with 1906I strain (MOI=25, 5 hpi). **(D)** Immunoblots for GSDME cleavage in HeLa or NCI-H226 cells infected with *E. piscicida* EIB202 or 1906I. HeLa cells treated with staurosporine (20 µM) as a positive control, cells without any treatment as a negative control. **(E, F)** Cell morphology with PI staining **(E)** and LDH release **(F)** of HeLa cells infected with *E. piscicida* EIB202 or 1906I (MOI=25, 5 hpi) in the presence of necrostatin-1 (Nec-1, 50 µM), GSK’872 (3 µM), necrosulfonamide (NSA, 20 µM), or DMSO. **(G)** LDH release of wild-type or *mlkl* ^-/-^ HeLa cells infected with *E. piscicida* EIB202 or 1906I at an MOI of 25 for the indicated time. Scale bar, 20 μm. Results are representative of at least three independent experiments, and error bars denote SD of triplicate wells. *P < 0.05 ***P < 0.001; n.s, not significant. [Two-way ANOVA for panels **(A, C, G)** One-way ANOVA for panels **(B, F)**].

Because 1906I-induced cell death showed both apoptotic and necrotic-like features, including membrane blebbing, swelling and membrane rupture, we assessed whether caspase-mediated apoptotic, pyroptotic cell death or necroptosis may be involved in the response to infection with 1906I. To identify the possible signaling pathway of host cell death induced by dysregulated c-di-GMP in *E. piscicida*, we firstly assessed the role of caspases by measuring the ability of cell lysates from HeLa cells to cleave a panel of fluorogenic and chromogenic caspase peptide substrates. As shown in **Figure 3A**, cell lysates from 1906I-infected HeLa cells did not show difference in their ability to cleave any of the caspase substrates tested when compared with those from EIB202-infected cells (**Figure 3A**). To further verify this, z-YVAD-fmk, z-VDVAD-fmk, z-DEVD-fmk, z-WEHD-fmk, z-VEID-fmk, z-IETD-fmk, z-LEHD-fmk, and z-VAD-fmk were used to inhibit the activity of caspase 1/4, caspase 2, caspase 3/7, caspase 5, caspase 6, caspase 8, caspase 9 and all caspases, respectively. Treatment with these panel of caspase inhibitors did not reduce cytotoxicity (**Figure 3B**) or morphological changes (**Figure S3A**) in 1906I-infected HeLa cells. These data suggest that 1906I-induced cell death is independent of caspases.

Several types of cell death mediated by caspases culminate in the formation of a lytic pore induced by the cleavage of pore-forming proteins Gasdermin D (GSDMD) and Gasdermin E (GSDME) (Shi et al., 2015; Rogers et al., 2017; Wang et al., 2017). To assess a role for GSDMD in cell death induced by the 1906I mutant, we infected wild-type and GSDMD-deficient HeLa cells with 1906I. However, GSDMD deficiency did not prevent the death of HeLa cells induced by 1906I infection (**Figure 3C**). Moreover, GSDME was not cleaved into the active N-terminal fragment either in HeLa cells or GSDME-highly expressing NCI-H226 cells upon *E. piscicida* strains infection while it was cleaved after treatment with the caspase 3 activator staurosporine (**Figure 3D**). We also assessed whether 1906I-induced cell death was induced *via* RIP1/RIP3-mediated necroptosis. We found that cytotoxicity and morphology induced by 1906I were not inhibited by the RIP1 inhibitor necrostatin-1, the RIP3 inhibitor GSK’872, or the MLKL oligomerization inhibitor necrosulfonamide (**Figures 3E, F**). In accord with these results, MLKL deficiency did not impair the cell death induced by 1906I (**Figure 3G**). Taken together, these data suggest that the upregulation of c-di-GMP in *E. piscicida* promotes non-pyroptotic and non-necroptotic membrane-ruptured cell death independent of caspases, Gasdermin D/E, RIP1/3, or MLKL.

### C-di-GMP-Mediated Cell Death Is Iron-Dependent

**Figure 4:**
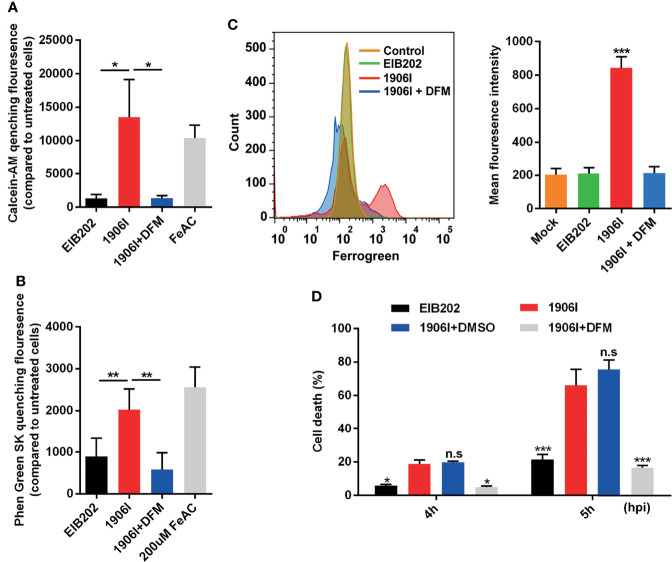
C-di-GMP-upregulated *E. piscicida* promoted cytosolic and mitochondrial labile iron accumulation triggering an iron-dependent cell death. **(A, B)** Calcein-AM **(A)** and Phen Green SK **(B)** quenching fluorescence intensity of HeLa cells treated with indicated *E. piscicida* strains or 200 µM ferric ammonium citrate (FeAC) with or without deferoxamine mesylate (DFM, 50 µM) for 3 h when compared to cells incubated with Opit-MEM only. Fluorescence intensity of cells was measured with a Multiscan Spectrum microplate reader and quenched fluorescence intensity compared to untreated cells reflected the cytosolic labile iron content. **(C)** Flow cytometric detection of mitochondrial labile iron in HeLa cells infected with *E. piscicida* EIB202 or 1906I with or without DFM (50 µM) by mitochondria-specific fluorescent probe Mito-FerroGreen. **(D)** LDH release of HeLa cells infected with *E. piscicida* EIB202 or 1906I at an MOI of 25 for the indicated time in the presence of DFM (50 µM) or DMSO. Results are representative of at least three independent experiments, and error bars denote SD of triplicate wells. *P < 0.05, **P < 0.01, ***P < 0.001; n.s, not significant. [One-way ANOVA for panels **(A–C)**, Two-way ANOVA for panel **(D)**].

Because lytic cell death induced by 1906I is neither pyroptosis nor necroptosis, we investigated whether the induced cell death was ferroptosis. To assess this possibility, we first examined whether there is intracellular iron accumulation in response to 1906I infection. The fluorescent probes CA-AM and PGSK, were used to quantitively detect cytosolic labile iron by measuring the quenched fluorescence intensity (Epsztejn et al., 1997; Petrat et al., 1999). Notably, there was an increase in cytosolic labile iron in HeLa cell infected with 1906I or incubation with FeAC that was used as a positive control when compared with EIB202 infection (**Figures 4A, B**). The increase in labile iron induced by 1906I infection was effectively inhibited by treatment with DFM, an iron chelator (**Figures 4A, B**). Because mitochondria are main destination and site of iron usage in cellular metabolism (Lv and Shang, 2018), we also tested the mitochondrial labile iron content after infection with 1906I. We found a marked increase in labile iron in the mitochondria of HeLa cells upon 1906I infection (**Figure 4C**). These results suggest that upregulation of c-di-GMP in *E. piscicida* promotes intracellular labile iron accumulation in host cells. Importantly, treatment with the iron chelator DFM suppressed the cell death induced by 1906I (**Figure 4D**). Taken together, these results suggest that c-di-GMP of *E. piscicida* induces cellular iron accumulation and triggers iron-dependent cell death after infection.

### Iron Accumulation Induces Mitochondrial Dysfunction and Oxidative Stress

**Figure 5:**
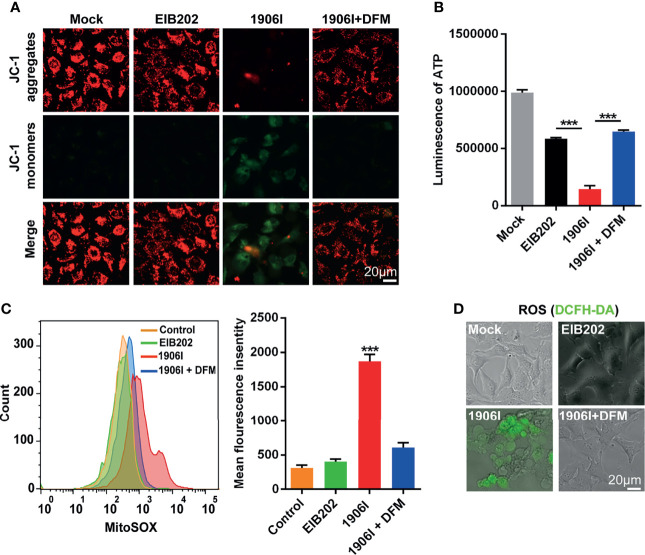
Iron accumulation induced decrease of mitochondrial potential and ATP production and enhanced mitochondrial and cytosolic ROS generation. HeLa cells were infected with indicated *E. piscicida* strains at an MOI of 25 with or without DFM (50 µM). **(A)** Mitochondrial membrane potential (ΔΨm) of HeLa cells was detected by JC-1 staining and observed by fluorescent microscopy at 3.5 h post bacterial infection. The double staining of cells by JC-1 is visible either as green for JC-1 monomers (low ΔΨm) or red for JC-1 aggregates (high ΔΨ). Scale bar, 20 μm. **(B)** ATP level of HeLa cells was measured by a luminescent ATP kit at 3.5 h post bacterial infection. **(C)** Flow cytometric detection of mitochondrial ROS by MitoSOX at 3.5 h post bacterial infection. **(D)** Determination of cellular ROS by DCFH-DA probe. Fluorescent microscopic images were taken at 3.5 h post bacterial infection. Scale bar, 20 μm. Results are representative of at least three independent experiments, and error bars denote SD of triplicate wells. ***P < 0.001 (One-way ANOVA).

Labile iron has high chemical redox reactivity which could catalyze excess Fenton reactions leading to the production of ROS and cytotoxicity (Winterbourn, 1995). Because mitochondria are major sites of oxygen consumption and electron transport, redox active labile iron in mitochondria could lead to oxidative stress and cell damage. To assess mitochondrial dysfunction, we labeled the cells with the mitochondrial membrane potential-sensitive dye JC-1 upon *E. piscicida* infection. We found a decrease of red fluorescence and an increase of green fluorescence as ΔΨm loss in HeLa cells infected with 1906I while the fluorescence in EIB202-infected cells remained unchanged and comparable to that observed in untreated cells (**Figure 5A**). The loss of mitochondrial potential induced by 1906I infection was inhibited by treatment with DFM (**Figure 5A**).

Because mitochondrial electron transport chain utilizes a series of electron transfer reactions to generate cellular ATP through oxidative phosphorylation (Nolfi-Donegan et al., 2020), we examined the effect of *E. piscicida* infection on ATP generation. Infection of HeLa cells with the 1906I mutant induced a reduction of ATP compared with infection with the wild-type EIB202 which was blocked by treatment with the iron chelator DFM (**Figure 5B**). We also investigated whether iron accumulation caused by 1906I also leads to intracellular oxidative stress. Compared with that observed after infection with EIB202, 1906I induced an increase of mitochondrial superoxide and intracellular ROS generation in HeLa cells, which was inhibited by addition of DFM (**Figures 5C, D**). Collectively, these results suggest that iron accumulation induces mitochondrial dysfunction and enhances cellular oxidative stress causing cell damage and cytotoxicity.

### C-di-GMP-Dysregulation Induces Non-Canonical Ferroptosis Independent of Lipid Peroxidation

**Figure 6:**
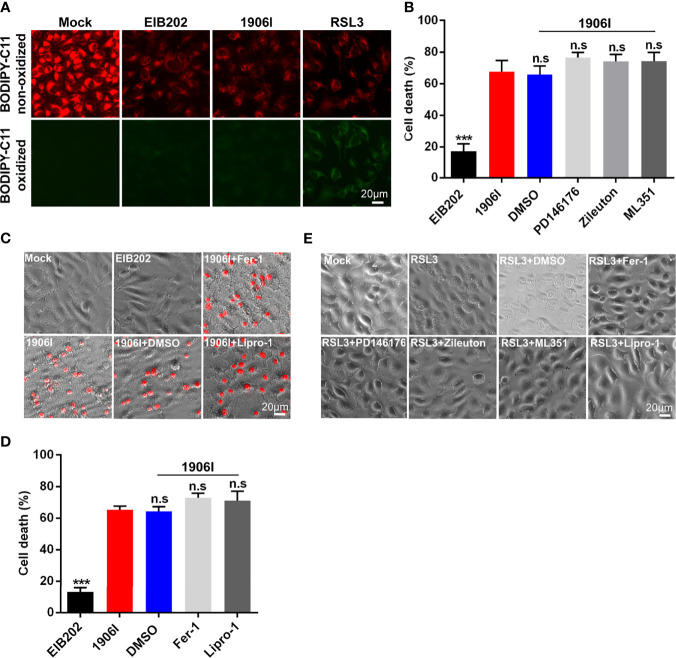
Cell death induced by c-di-GMP-upregulated *E. piscicida* was independent of lipid peroxidation. **(A)** Detection of lipid peroxidation by BODIPY-C11 in HeLa cells treated with indicated *E. piscicida* strains (MOI=25) or RSL3. Fluorescent microscopic images were taken at 3.5 h post bacterial infection or RSL3 treatment. Untreated cells as a negative control and RSL3-treated cells as a positive control. Scale bar, 20 μm. **(B)** LDH release of HeLa cells infected with *E. piscicida* EIB202 or 1906I (MOI=25, 5 hpi) in the presence of PD146176 (5 µM), zileuton (5 µM), ML351 (10 µM), or DMSO. **(C, D)** Cell morphology with PI staining **(C)** and LDH release **(D)** of HeLa cells infected with *E. piscicida* EIB202 or 1906I (MOI=25, 5 hpi) in the presence of ferrostatin-1 (Fer-1, 20 µM), liproxstatin-1 (Lipro-1, 20 µM), or DMSO. Scale bar for the images, 20 μm. **(E)** Cell morphology of RSL3-treated HeLa cells with PD146176 (5 µM), zileuton (5 µM), ML351 (10 µM), Fer-1 (20 µM), lipro-1 (20 µM), or DMSO. Scale bar, 20 μm. Results are representative of at least three independent experiments, and error bars denote SD of triplicate wells. ***P < 0.001; n.s, not significant. (One-way ANOVA).

Classical ferroptosis is an iron-dependent oxidative programed cell death characterized by the accumulation of lipid peroxides. Because of the iron-dependent and oxidative features of ferroptosis induced by 1906I infection, we next assessed whether 1906I triggers ferroptosis through lipid peroxidation. To assess this question, we utilized the lipid peroxidation sensor BODIPY-C11 whose fluorescence shifts from red to green upon oxidation in HeLa cells infected with *E. piscicida* strains. A ferroptosis inducer RSL3 induced ferroptosis in HeLa cells (**Figure 6E**) and an increase of green fluorescence was detected in cells stained with BODIPY-C11 (**Figure 6A**). In contrast, the fluorescence profile of cells infected by either 1906I or EIB202 was comparable to that observed in untreated cells (**Figure 6A**). These results suggest that *E. piscicida* infection with wild-type and mutant strains does not induce detectable lipid peroxidation under conditions in which the 1906I mutant induces iron-dependent cell death.

To further confirm these results, we treated HeLa cells with inhibitors of LOXs, which catalyze the deoxygenation of free and esterified polyunsaturated fatty acids to generate various lipid hydroperoxides (Hassannia et al., 2019). Treatment with PD146176, zileuton, and ML351 effectively inhibited the morphological change and cell death triggered by RSL3 (**Figure 6E**), but failed to suppress cytotoxicity caused by 1906I infection (**Figure 6B**). Because lipid peroxides can be generated not only by LOXs-catalyzed lipid peroxidation, but also by non-enzymatic or auto-oxidation of lipids (Hassannia et al., 2019), we treated the cells with lipophilic antioxidants Fer-1 and Lipro-1 to assess their effects on cytotoxicity caused by 1906I. Fer-1 and Lipro-1 suppressed RSL3-induced ferroptosis (**Figure 6E**), but they were unable to inhibit morphological changes or cytotoxicity in 1906I-infected HeLa cells (**Figures 6C, D**). Collectively, these results suggest that dysregulated c-di-GMP in *E. piscicida* promotes cell damage and death through a non-canonical ferroptosis pathway that is independent of lipid peroxidation.

## Discussion

C-di-GMP is a universal bacterial secondary messenger that is synthesized from two GTP molecules by DGC and degraded by phosphodiesterase (Romling et al., 2013). The cytosolic c-di-GMP content is tightly controlled by DGCs and phosphodiesterases and regulates bacterial function by binding to downstream effectors in response to environmental cues (Romling et al., 2013; Hall and Lee, 2018). High levels of c-di-GMP facilitate the synthesis of adhesins and biofilm matrix components including EPS and interfered with motility and virulence functions while decreased c-di-GMP is associated with opposite phenotypes (Hengge, 2009). As an important secondary messenger widely distributed in diverse bacteria, increasing evidence suggest that c-di-GMP plays a role in bacterial pathogenesis. For example, low intracellular c-di-GMP levels promotes expression of virulence factors in *Vibrio cholerae* while elevated intracellular c-di-GMP attenuates virulence in a neonatal mouse model of cholera infection (Tischler and Camilli, 2005). Likewise, deletion of *bpdA* and *bpdB*, which exhibited the cyclic-di-GMP phosphodiesterases phenotype in *Brucella melitensis* reduced its virulence in mice while deletion of *CgsB* displaying c-di-GMP synthase phenotype increased virulence (Petersen et al., 2011). High c-di-GMP levels also suppress acute infections of *Yersinia pestis* and *Borrelia burgdorferi* by upregulating the production of extracellular biofilm matrix (Bobrov et al., 2011; Sultan et al., 2011). Here, we revealed that transposon insertion in *E. piscicida* 1906I resulted in activation of DGC, causing an increase of cytosolic c-di-GMP, and enhanced production of EPS, biofilm formation, and bacterial adhesion as well as increased cytotoxicity in eukaryotic cells which blocked by inhibitors of c-di-GMP synthesis. We found that elevated c-di-GMP attenuated *E. piscicida*’s virulence in zebrafish which was correlated with reduced bacterial colonization in several organs early in infection. These results provide support for a contribution of c-di-GMP signaling to bacterial pathogenesis and in particular *E. piscicida* infection. However, further investigations are needed to figure out the specific mechanism of c-di-GMP signaling in regulating bacterial infection including what and how specific ci-di-GMP-regulated component affects eukaryotic cellular events since it remains to be possible that the DGC overexpression in 1906I might cause these phenotypes through a non-c-di-GMP pathway.

**Figure 7:**
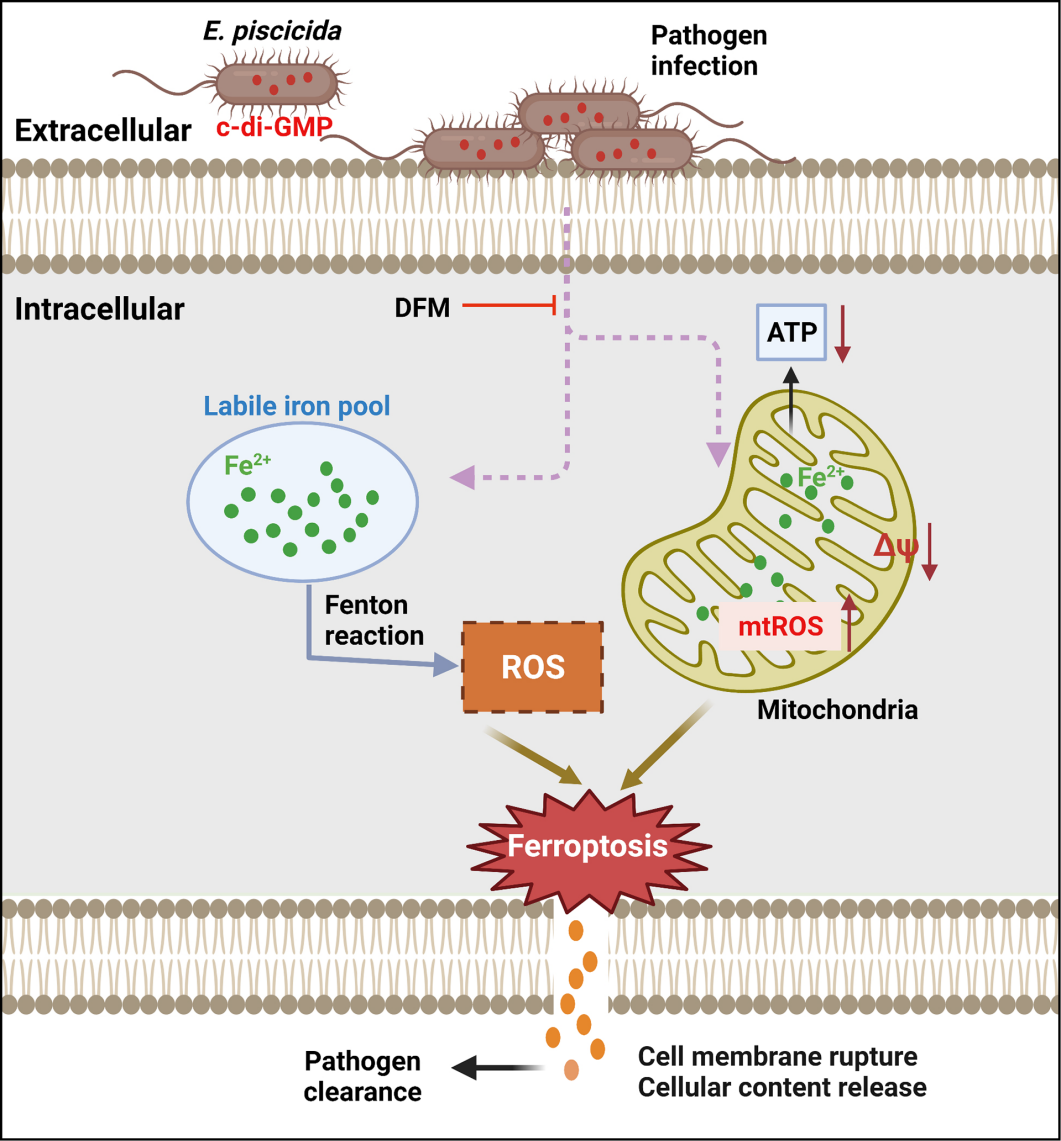
Summary of proposed mechanism of bacterial c-di-GMP promoting non-canonical ferroptosis. Elevated c-di-GMP in *E. piscicida* promotes cellular labile iron accumulation resulting in decrease of mitochondrial potential and ATP production, and increase of mitochondrial and cytosolic ROS generation triggering a non-canonical ferroptosis which is independent of lipid peroxidation and could be inhibited by iron chelator DFM. The dysregulation of c-di-GMP in *E. piscicida* also attenuates its colonization and virulence *in vivo*.

Ferroptosis has been reported to be catalyzed and initiated by iron through accelerating Fenton reaction to generate ROS, and executed by excessive peroxidation of polyunsaturated fatty acids in cell membranes (Dixon et al., 2012; Hassannia et al., 2019). Ferroptosis-inducers RSL3 and Erastin can directly or indirectly affect glutathione peroxidase 4 resulting in a decrease of antioxidant capacity and accumulation of lipid ROS ultimately leading to cell death (Dixon et al., 2012; Li et al., 2020). Accordingly, ferroptosis can be blocked by iron chelators or lipid peroxides-related inhibitors including LOXs inhibitors and lipophilic antioxidants (Dixon et al., 2012; Yang et al., 2014; Hassannia et al., 2019; Li et al., 2020). Morphologically, cells undergoing ferroptosis have a typical necrotic morphology along with small aberration and decreased potential of mitochondria, but no clear hallmarks of apoptosis (Li et al., 2020). An important finding of our work is the link of dysregulation of c-di-GMP to lytic cell death in host cells infected with *E. piscicida*. We found that increased production of c-di-GMP by *E. piscicida* triggers ferroptosis (**Figure 7**). However, unlike classical ferroptosis or other regulated necrotic cell death, morphologically, cells infected with *E. piscicida* 1906I also exhibited apoptotic features including membrane blebbing and formation of balloon-like protrusions. In addition, unlike classical ferroptosis, we found no evidence for a role of lipid peroxidation in ferroptosis induced by 1906I infection. The results were further validated by using diverse LOXs inhibitors and lipophilic antioxidants, all of which failed to inhibit morphological changes and cell death in response to infection with *E. piscicida* 1906I. Thus, our studies suggest that cell death induced by the 1906I mutant represents a non-canonical ferroptosis pathway that is independent of lipid peroxidation. Although the iron-dependent mediators that are responsible for non-canonical ferroptosis in our system remain unclear, it is possible that non-lipid toxic molecules or different types of lipids could induce oxidative stress and cell damage. Additional work is needed to understand how dysregulated c-di-GMP induces iron accumulation and the iron-dependent molecules that mediate lytic cell death.

Previous studies of ferroptosis mostly focused on its role in sterile disease such as neurodegeneration, acute kidney injury and cancer, but few studies have addressed the relevance of ferroptosis during pathogen infection. A recent study found that *Pseudomonas aeruginosa* can utilize lipoxygenase (pLoxA) to oxidize host arachidonic acid-phosphatidylethanolamines (AA-PE) to 15-hydroperoxy-AA-PE triggering ferroptosis in human bronchial epithelial cells (Dar et al., 2018). *Mycobacterium tuberculosis* was also reported to induce accumulation of labile iron and lipid peroxidation in macrophages both *in vitro* and *in vivo* (Amaral et al., 2019). In the present work, we found that upregulation of c-di-GMP in *E. piscicida* strain can also lead to increased labile iron, mitochondrial superoxide inducing cell death which can be blocked by iron chelator in HeLa cells. However, unlike *Pseudomonas. aeruginosa* or *Mycobacterium tuberculosis*, *E. piscicida* induced a non-canonical ferroptosis which is independent of lipid ROS and cannot be suppressed by Fer-1 or other lipophilic antioxidants. Moreover, animals infected with *Mycobacterium tuberculosis* display prominent decrease in bacterial loads when ferroptosis is suppressed (Amaral et al., 2019). In contrast, *E. piscicida*-induced ferroptosis correlated with protective function in the host during infection in that zebrafish infected with the mutant *E. piscicida* 1906I showed reduced pathogen loads and increased survival. These differences could be explained by the use of a different model of pathogen and host infection, differences in the ferroptosis pathway or the fact that in our model ferroptosis was trigger by c-di-GMP dysregulation in the mutant 1906I.

Collectively, our study shows that elevated c-di-GMP in *E. piscicida* triggers an iron-dependent, non-canonical ferroptosis, which is independent of lipid peroxidation, and reduces pathogen virulence in zebrafish (**Figure 7**), indicating the importance of tight regulation of intracellular c-di-GMP during infection and presenting a non-canonical ferroptotic pathway independent of lipid peroxidation that can be triggered by pathogen infection.

## Data Availability Statement

The original contributions presented in the study are included in the article/**Supplementary Material**. Further inquiries can be directed to the corresponding author.

## Ethics Statement

The animal study was reviewed and approved by Institutional Animal Care and Use Committee of East China University of Science and Technology (No. 2006272).

## Author Contributions

QL conceived and supervised the study. YWen conducted most of the experiments with the help of YWang and SC. YWen and DY wrote the manuscript. GN, XZ, and YZ critically revised the manuscript. All authors discussed the results and commented on the manuscript. All authors contributed to the article and approved the submitted version.

## Funding

This work was supported by Frontier Science Research Base of Optogenetic Techniques for Cell Metabolism grant 2021 Sci & Tech 03-28 (Shanghai Municipal Education Commission), National Natural Science Funds for Distinguished Young Scholar (No. 32025038), and China Postdoctoral Science Foundation (No. 2020M671033). YWen was supported by the State Scholarship Fund from China Scholarship Council (No. 201906740067).

## Conflict of Interest

The authors declare that the research was conducted in the absence of any commercial or financial relationships that could be construed as a potential conflict of interest.

## Publisher’s Note

All claims expressed in this article are solely those of the authors and do not necessarily represent those of their affiliated organizations, or those of the publisher, the editors and the reviewers. Any product that may be evaluated in this article, or claim that may be made by its manufacturer, is not guaranteed or endorsed by the publisher.
