# Supplementary material for: Dysregulation of Cytosolic c-di-GMP in Edwardsiella piscicida Promotes Cellular Non-Canonical Ferroptosis
Source: Front Cell Infect Microbiol. 2022 Feb 4;12:825824. doi: 10.3389/fcimb.2022.825824 (PMC8855483; doi:10.3389/fcimb.2022.825824)
Supplement: Supplementary file 1 [file DataSheet_1.pdf]

Supplementary Material

Supplementary Figures

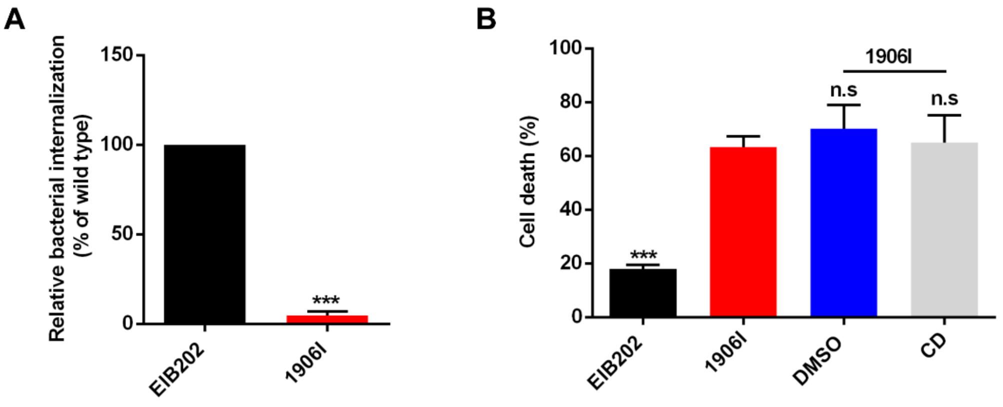

Supplementary Figure 1. 1906I-induced cell death is not dependent on bacterial internalization. (A) Bacterial count by agar plating cell lysates HeLa cells infected with *E. piscicida* EIB202 or 1906I (MOI=100, 1 hpi) after treatment with 800 µg/ml streptomycin for 0.5 h to kill extracellular bacteria. (B) LDH release of HeLa cells infected with *E. piscicida* EIB202 or 1906I (MOI=25, 5 hpi) in the presence of cytochalasin D (2 µg/ml) or DMSO. Results are representative of at least three independent experiments, and error bars denote SD of triplicate wells. ***$P < 0.001$; n.s, not significant. [Unpaired *t* test for panel (A) and one-way ANOVA for panel (B)].

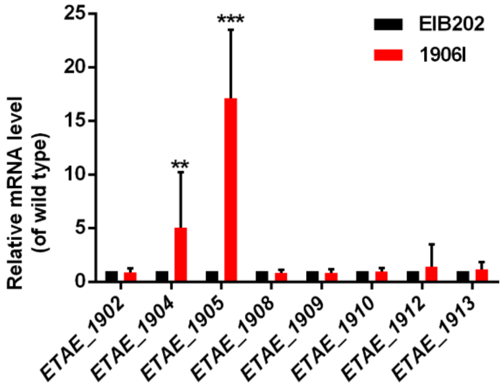

Supplementary Figure 2. **Quantitative PCR for mRNA of genes in *E. piscicida* genome adjacent to transposon insertion site**. Results are representative of at least three independent experiments, and error bars denote SD of triplicate wells. $^{**}P < 0.01$, $^{***}P < 0.001$. (Two-way ANOVA).

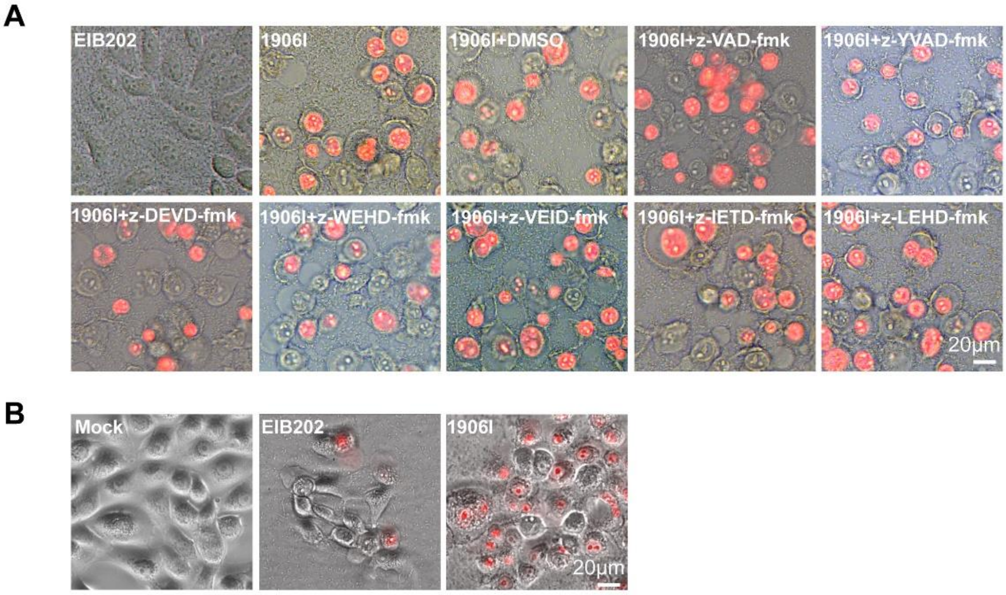

Supplementary Figure 3. Morphology analysis of HeLa cells infected with *E. piscicida* strains. (A) Morphology of HeLa cells infected with *E. piscicida* EIB202 or 1906I (MOI=25, 5 hpi) in the presence of z-VAD-fmk (50 μM), z-YVAD-fmk (50 μM), z-VDVAD-fmk (50 μM), z-DEVD-fmk (50 μM), z-WEHD-fmk (20 μM), z-VEID-fmk (50 μM), z-IETD-fmk (50 μM), z-LEHD-fmk (50 μM), or DMSO. (B) Morphology of NCI-H226 cells infected with *E. piscicida* EIB202 or 1906I (MOI=25, 5 h). Propidium iodide (PI) was added to detect the loss of plasma membrane integrity. Scale bar, 20 μm.

Supplementary Table

Supplementary Table 1. Primers used for *E. piscicida* mutants' construction

**Primers**	**Sequence (5'- 3')**
depletion-*ETAE_1906*-P1	GTTACCCGGATCTATCTAGA
	TAGAGCCGTATCATGCACTG
depletion-*ETAE_1906*-P2	TTTCACCCTGTGTGACATTCATGCAGCAGG
depletion-*ETAE_1906*-P3	GAATGTCACACAGGGTGAAACGCTTATT
depletion-*ETAE_1906*-P4	AGCTTATCGATACCGTCGAC
	GCCCCAGCAGCAGATAGCCCAAGAA
depletion-*ETAE_1905*-P1	CCCCCCCGAGCTCAGGTTACCCGGATCTAT
	TACGGCGCCTGGATCACGAT
depletion-*ETAE_1905*-P2	TGGCAAAGAAAAAAGGGTAA
	ATGCCGCCATGTATCGCAGT
depletion-*ETAE_1905*-P3	ACTGCGATACATGGCGGCAT
	TTACCCTTTTTTCTTTGCCA
depletion-*ETAE_1905*-P4	GAGTACGCGTCACTAGTGGGGCCCTTCTAG
	TGGGCATACGGGAAGAAGTG

Supplementary Table 2. Primers for real-time quantitative PCR (RT-qPCR) analysis

**Gene name**	**Forward Primers (5'- 3')**	**Reverse Primers (5'- 3')**
ETAE_1902	CAATGCGCAGACAATTCAGC	TGATCCTTATAGCCGCCCAG
ETAE_1904	TACAACAACGAAAGGGCTGG	TACAACAACGAAAGGGCTGG
ETAE_1905	CGGCCCTATCGAACACAATG	CCCGTAATGAATAGGTGCGC
ETAE_1906	GCATTATTCCACCGACGCAT	GATCGTCAGCAGGCCTTTTC
ETAE_1908	GATCCAGTGCATGATACGGC	AGTGGATTGGACAGCGTTTG
ETAE_1909	GGCGGAAGAGTGCATGAATA	GAGCCAACGACGATCAGATC
ETAE_1910	AATATGTGGCCTGGGTAGCA	GGTGGTTGATGGTGGTAGGA
ETAE_1912	CTGGTGGTGGAGGACAATCT	ATCCATCAGCACCAGGTCAA
ETAE_1913	GTTTATCTGTCTGCTGCCGG	TGTGACTTGGCCTGGTAGAG
gyrB	ATTGGCCGGGATGAGTACAA	ATCCATCGCCTCATCGTCTT
